# In Vitro Study of the Anticancer Effects of Biotechnological Extracts of the Endangered Plant Species *Satureja Khuzistanica*

**DOI:** 10.3390/ijms20102400

**Published:** 2019-05-15

**Authors:** Abbas Khojasteh, Isidoro Metón, Sergio Camino, Rosa M. Cusido, Regine Eibl, Javier Palazon

**Affiliations:** 1 Secció de Fisiologia i Biotecnologia Vegetal, Departament de Biologia, Sanitat i Medi Ambient, Facultat de Farmàcia i Ciències de l’Alimentació, Universitat de Barcelona, Joan XXIII 27-31, 08028 Barcelona, Spain; abbaskhojasteh@aol.com (A.K.); rcusido@ub.edu (R.M.C.); 2 Secció de Bioquímica i Biologia Molecular, Departament de Bioquímica i Fisiologia, Facultat de Farmàcia i Ciències de l’Alimentació, Universitat de Barcelona, Joan XXIII 27-31, 08028 Barcelona, Spain; imeton@ub.edu (I.M.); scamino93@gmail.com (S.C.); 3 Institute of Chemistry and Biotechnology, Biochemical Engineering and Cell Cultivation Techniques, Campus Grüental, Zurich University of Applied Sciences, 8820 Wädenswill, Switzerland; eibs@zhaw.ch

**Keywords:** *Satureja khuzistanica*, rosmarinic acid, plant cell cultures, coronatine, MCF-7 human breast adenocarcinoma cells, HepG2 human hepatoma cells

## Abstract

Many medicinal plant species are currently threatened in their natural habitats because of the growing demand for phytochemicals worldwide. A sustainable alternative for the production of bioactive plant compounds are plant biofactories based on cell cultures and organs. In addition, plant extracts from biofactories have significant advantages over those obtained from plants, since they are free of contamination by microorganisms, herbicides and pesticides, and they provide more stable levels of active ingredients. In this context, we report the establishment of *Satureja khuzistanica* cell cultures able to produce high amounts of rosmarinic acid (RA). The production of this phytopharmaceutical was increased when the cultures were elicited with coronatine and scaled up to a benchtop bioreactor. *S. khuzistanica* extracts enriched in RA were found to reduce the viability of cancer cell lines, increasing the sub-G0/G1 cell population and the activity of caspase-8 in MCF-7 cells, which suggest that *S. khuzistanica* extracts can induce apoptosis of MCF-7 cells through activation of the extrinsic pathway. In addition, our findings indicate that other compounds in *S. khuzistanica* extracts may act synergistically to potentiate the anticancer activity of RA.

## 1. Introduction

Rosmarinic acid (RA), an ester of caffeic acid and 3,4-dihydroxyphenyl lactic acid, is widely distributed in the plant kingdom, including the genera *Ajuga*, *Agastache, Calamintha, Cedronella, Coleus, Collimsonia, Dracocephalum, Elsholtzia, Glechoma, Hornium, Lavandula, Lycopus, Melissa, Mentha, Micromeria, Monarda, Origanum, Perilla, Perovskia, Plectranthus*, and *Salvia* from the Lamiaceae family, *Cerinthe, Echium, Heliotropium, Lindefolia, Lithospermum, Nonea, Symphytum, Hydrophyllum, Nemophila* and *Phacelia* from the Boraginaceae family, *Chlorantus* from the Chloranteaceae family and *Blechnum* from the Blechnaceae subfamily [1,2].

Interest in RA has grown due to increasing awareness of its potential benefits for human health as a pharmaceutical or dietary supplement. Among its promising biological activities are cognitive-enhancing and cardioprotective effects, cancer chemoprevention properties, and a potential use in the treatment of Alzheimer’s disease [3].

Among diseases affecting our society, cancer is one of the most widespread and has the highest mortality rate. Thus, the search for new antineoplastic agents and the confirmation of recently discovered action mechanisms is a major challenge for medicine. In a review of the biological effects of RA, Moore et al. [4] concluded that it could be used as a phytochemical to induce apoptosis. RA can reduce survival of cancer cell lines such as HT-28 (colorectal adenocarcinoma), MCF-7 (breast carcinoma), DU145 (prostate carcinoma) or MKN45 (gastric carcinoma), among others. In the same pipeline, RA, which is currently exploited as an antioxidant and food additive, could also function as a nutraceutical to enhance the effects of other chemotherapeutics. 

As a result of overexploitation, many species of medicinal plants are now under threat of extinction in their original habitats. This is the case of *Satureja khuzistanica*, an endangered native Iranian plant that accumulates up to 1.81% of RA [5]. When the natural source of a phytochemical cannot meet the market demand, or is becoming increasingly limited because of over-harvesting or habitat deterioration, plant biotechnology can provide an alternative production system. Plant cell cultures producing phytochemicals have several advantages over field cultivation: (a) The target product can be harvested anywhere in the world, while maintaining strict production and quality control; (b) herbicides and pesticides are not required; (c) problems related with climate and ecology are avoided; and (d) growth cycles are significantly reduced compared to the intact plant, taking weeks rather than years [6]. 

In this scenario, our group has recently been developing a biotechnological platform for the production of RA based on plant cell cultures of *S. khuzistanica* [7]. In optimal conditions, plant cells cultured in shake flasks and elicited with 100 µM methyl jasmonate reached an RA production of 245 mg·g·DW^−1^ after 16 days of culture. Moreover, when the system was scaled up to a wave-mixed bag of the BIOSTAT CultiBag RM (working volume of 1 L) running in batch mode for a growth period of 21 days, the RA productivity was 3601 mg·L^−1^, which demonstrated the suitability of the process for the commercial production of RA.

In the current work, with the aim of further improving the RA productivity of this culture system, we studied the action of the new elicitor coronatine (COR), which acts as a molecular mimic of the isoleucine-conjugated form of jasmonic acid [8]. We also tested the anticancer activity of biotechnologically produced *S. khuzistanica* extracts by analyzing their effects on the viability of MCF-7 and HepG2 cancer-derived cell lines, as well as the cell cycle and caspase activity of MCF-7 cells.

## 2. Results and Discussion

### 2.1. Effect of Coronatine on a Small Scale

Elicitation with COR has been shown to have a positive impact on intracellular taxane accumulation in in vitro cultures of Taxus spp. [9]. However, the eliciting effects of COR on metabolite production depend on the concentration used and time of exposure, the plant species and cell line.

**Figure 1:**
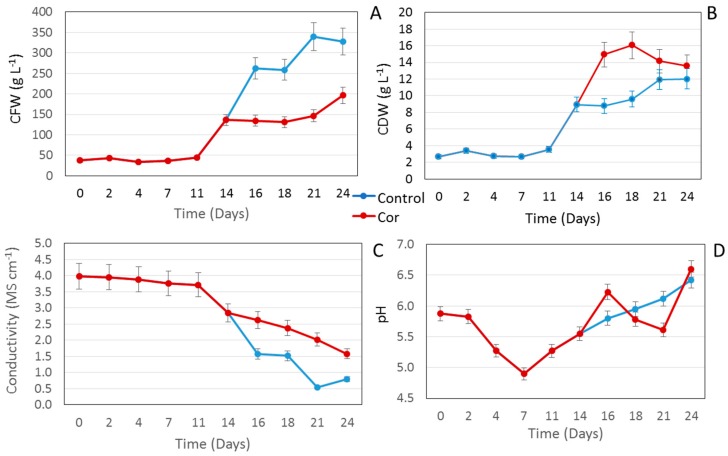
Time course of changes in cell fresh weight (CFW) (**A**), cell dry weight (CDW) (**B**) expressed as g·L^−1^, conductivity (**C**) and pH (**D**), over a growth period of 24 days. Each result is the average of 3 replicates ± SD.

To optimize the growth capacity of the *S. khuzistanica* cell suspension cultures, a starting packed cell volume (PCV) of 10% was prepared under previously established optimal conditions [7]. As shown in Figure 1A, after more than a week in the lag phase (until day 11), growth measured as cell fresh weight (CFW) shifted to the exponential phase, which lasted until day 16. Thereafter, a slight increase of CFW was observed and at day 18 the cultures entered the stationary phase, reaching a final biomass of 339.5 g·L^−1^ in control conditions. 

The growth capacity of the suspension cultures was significantly reduced (*p* < 0.05) by the addition of COR on day 14. A similar effect was reported for methyl jasmonate (MeJA) in the same cell line [7], but the reduction in biomass was considerably less (1%) compared to COR (nearly 10%), indicating the latter is more toxic. These results differ from those obtained with Taxus cell cultures, in which COR was more effective than MeJA as an inducer of taxol production without significantly affecting the growth capacity of the cultures [10].

The time course of growth measured as cell dry weight (CDW) was very similar to that of the CFW. The lag phase also lasted until day 11 and the CDW significantly decreased in the COR-treated cultures compared to the control during the exponential growth phase, being significantly lower at the end of the culture period (Figure 1B). Thus, whether measured by CFW or CDW, the growth capacity of *S. khuzistanica* cell suspension cultures was significantly reduced by the elicitor.

A comparison of the time course of growth measured as CFW and CDW (Figure 1A,B) with previous growth curves obtained by Khojasteh et al. [7] with the same *S. khuzistanica* cell culture revealed that the cell line growth capacity had changed over time and after subculturing, even in control conditions. Differences in growth between different subcultures could be attributed to small variations in the culture conditions and the inherent changes associated with the culture age [11,12,13]. In this context, our group has recently demonstrated that epigenetic modifications appear in cell lines with successive subcultures, which can affect not only the growth capacity but also secondary metabolite production [14]. This could also have occurred in our *S. khuzistanica* cell line, although an epigenetic study of the degree of DNA methylation is required to prove this hypothesis.

Cells absorb nutrients from the medium, including the macro- and microelements that are added as ions/salts. Thus, when cells grow, the concentration of ions decreases, which in turn reduces the medium conductivity [15]. In our study, the conductivity decreased continuously throughout the experiment. Under the control conditions it dropped significantly at day 16 (exponential growth phase), whereas in the COR-treated suspension cultures the decline on this day and thereafter continued to be slight (Figure 1C). This difference was probably due to the higher consumption of medium salts in control cultures and the lower growth capacities of the treated cultures. In other words, the increase in CFW and CDW in control cultures inversely correlated with nitrate consumption and the decrease in conductivity.

The cell suspension cultures were initiated at a pH of 5.8, which dropped to 4.9 at day 7, possibly as a result of the high uptake of ammonium. When the exponential growth phase began, the pH started to increase again, probably due to the nitrate uptake. COR had a clear effect on the pH, which increased after the day of elicitation (day 14). The pH then decreased gradually until day 21, before increasing again when the cells entered the death phase (Figure 1C).

The specific production of RA in *S. khuzistanica* suspension cultures was determined every two days throughout the experiment and expressed as mg·g·CDW^−1^ and in mg·L^−1^. The presence of RA was confirmed in all the *S. khuzistanica* methanolic extracts, as shown in Supplementary Figure S1, which depicts the corresponding UV chromatogram (330 nm) with a main peak for RA at a retention time of 16 min. In control conditions (untreated cells), the specific production of RA increased throughout the culture period, reaching a final content of 164 mg·g·CDW^−1^ (Table 1). The addition of COR significantly enhanced the accumulation of RA (Table 1). In the treated cells, the RA production reached a maximum of nearly 221 mg·g·CDW^−1^ between days 4 and 10 after elicitation. At day 18 (96 h after elicitation), the RA content was about 1.5-fold higher than in the control cells. 

The RA production of the biotechnological system was also expressed as mg·L^−1^ of culture volume (Table 1), which takes into consideration the biomass-producing capacity of the system, unlike when expressed as mg·g·CDW^−1^. As COR significantly reduced the CDW, the productivity of RA also decreased significantly after the elicitation (Table 1). However, at day 21 (168 h after elicitation), the RA production levels of COR-treated cells started to overtake those of the control cells, reaching more than 2600 mg·L^−1^ after 240 h, which was significantly (*p* > 0.05) higher than the control. 

In previous experiments [7], MeJA elicitation of cells resulted in a maximum RA production of about 3858 mg·L^−1^ at day 16, whereas the maximum production with COR was reached at day 24 (Table 1). In the study by Khojasteh et al. [7], production in control conditions peaked at day 14 (1436 mg·L^−1^), compared to day 24 (2221 mg·L^−1^) in the current work, indicating that age affects the production rate and growth course of the cell line. In contrast with studies on other plant species, which lost the capacity to produce secondary metabolites with age, production in our *S. khuzistanica* cell line did not decrease with time [13,14,16].

### 2.2. Scaling Up the Process to a Benchtop Bioreactor

**Figure 2:**
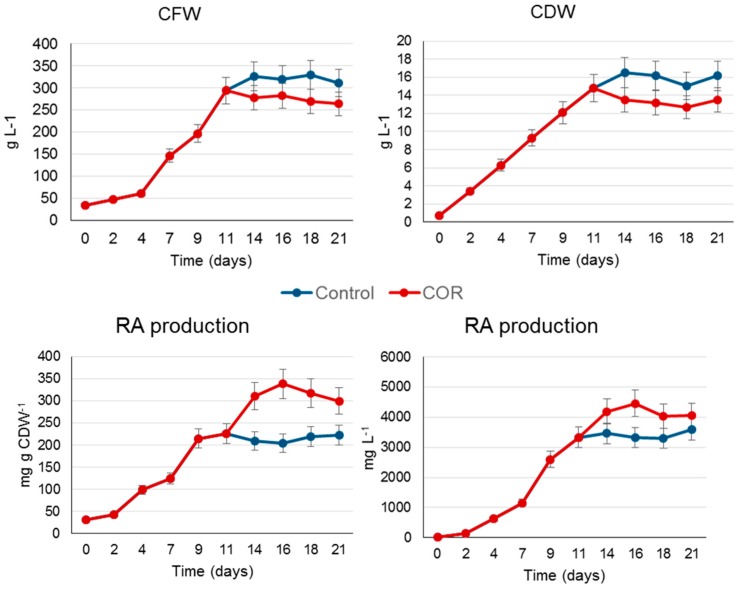
Biomass measured as cell fresh weight (CFW) and dry cell weight (CRW), and rosmarinic acid (RA) production in *S. khuzistanica* control and COR-elicited cultures in a benchtop bioreactor during a growth period of 21 days. The data are the average of two replicates ± SD.

**Figure 3:**
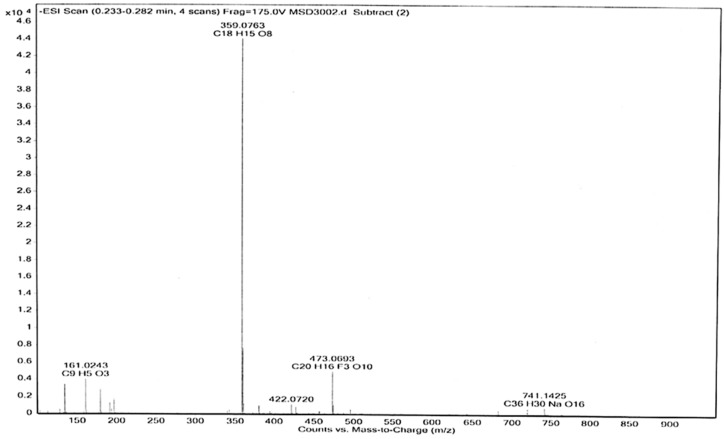
Electrospray ionization (ESI-MS) base peak chromatogram of a rosmarinic acid (RA)-enriched methanolic extract of *S. khuzistanica* cell cultures (SKE) in negative ion mode. Mass spectra were detected in the range of m/z 100–1000. A molecular weight of 359 was assigned to [M−H]^−^, and 741 to [2M−2H+Na]^−^ of RA.

The elicitation assay was scaled up to a 2 L culture bag (CellBag). This type of power input and bioreactor are suitable for the culture of mammalian and plant cells with a low oxygen demand [17]. The time course of the biomass production measured as CFW showed a typical growth curve with a lag phase of 2–4 days, followed by an exponential growth phase that finished at day 14, when the culture entered the stationary phase. Thereafter, the CFW did not increase for the remainder of the culture period (up to day 21) (Figure 2A). A similar curve was obtained when the growth was measured as CDW, although in this case the lag phase of the culture was not apparent (Figure 3B). In control conditions (untreated cells), the lag phase in the bioreactor was clearly shorter than in the shake flask experiment (Figure 1 and Figure 2). In contrast, under elicitation, the negative effect of COR on growth capacity was lower in the bioreactor cultures (Figure 1 and Figure 2).

As in the shake flasks (Table 1), COR significantly increased (*p* > 0.05) the RA production capacity of the bioreactor system, achieving a specific production of 338.2 mg·g·CDW^−1^ at day 16, which was 1.7 times higher than in control conditions (untreated cells) (Figure 3). When measured as mg·L^−1^ of culture medium, the RA production pattern was similar, and from day 14 the yield was higher in the COR-treated cultures (Figure 2). Notably, the cultures grew better in the orbitally shaken bag than in shake flasks and the negative effects of COR on biomass production were much less apparent. This fact, together with a high specific RA production (mg·g·CDW^−1^) in bioreactor conditions, resulted in yields that were 2.3-fold higher at day 16 than in the small-scale system. 

Taken as a whole, these results demonstrate the suitability of the orbitally shaken CellBag for scaling up the suspension cultures of *S. khuzistanica*. Similar results have been achieved with *Centella asiatica* cell cultures, where growth and centelloside production also improved at bioreactor level [18]. Comparison of biomass and RA production of the *S. khuzistanica* cell line obtained in the wave-mixed and the orbitally shaken bag [7] revealed that both bioreactor systems are effective. In all cases, the growth rate was higher at bioreactor level than in shake flasks, as was the case for maximum RA production (Figure 2). However, if we compare the final yield achieved in control conditions (un-elicited cells) in both types of bioreactor, it was higher (3600 mg·L^−1^) in the orbitally shaken bag than in the wave-mixed bag (3102 mg·L^−1^). This points to different degrees of adaptation of the *S. khuzistanica* cell cultures to the two systems, but as mentioned, the two experiments were not developed simultaneously, and as reported in other studies, the age of a cell culture can have a dramatic effect on the system productivity [13,16].

### 2.3. Reduction of MCF-7 Cell Viability by S. Khuzistanica Extracts

The predominance of RA in the methanolic *S. khuzistanica* extract (SKE), obtained as described in Section 3.5 (see Supplementary Figure S1), was confirmed by electrospray ionization (ESI-MS) in positive and negative mode (Figure 3). The effect of SKE on cell viability was studied in MCF-7 and HepG2 cells, either non-treated or incubated for 48 h with different amounts of SKE, RA (positive control known to reduce cell viability) or vehicle (dimethyl sulfoxide, DMSO).

**Figure 4:**
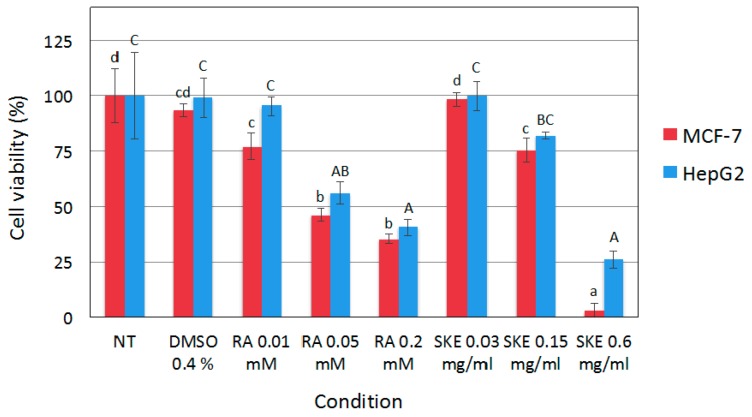
Effect of *S. khuzistanica* extracts on viability of MCF-7 and HepG2 cells. Cell viability was assayed 48 h following treatment with 0.03 mg·mL^−1^, 0.15 mg·mL^−1^ and 0.6 mg·mL^−1^ *S. khuzistanica* extract (SKE), 0.01 mM, 0.05 mM and 0.2 mM rosmarinic acid (RA) or 0.4% DMSO (vehicle; D). Control cells were non-treated (NT). Each bar represents the mean ± SD of three replicates. Different letters (lower case for MCF-7 cells and upper case for HepG2 cells) indicate significant differences between treatments (*p* < 0.01).

As shown in Figure 4, the use of up to 0.4% (*v*/*v*) of DMSO as a vehicle did not significantly affect MCF-7 and HepG2 cell viability. Therefore, DMSO was selected as the solvent for subsequent experiments at concentrations of up to 0.4%. The highest concentration of SKE assayed (0.6 mg·L^−1^) significantly reduced the viability of HepG2 cells up to about 25% of the values observed in control cells, and decreased MCF-7 cell viability to barely detectable levels. A similar cytotoxic effect on MCF-7 and HepG2 cells was observed with the highest concentration of RA used (0.2 mM). Remarkably, even though RA content in 0.6 mg·L^−1^ SKE was estimated to be 0.064 mM, the biotechnologically produced extract had a stronger effect on MCF-7 and HepG2 cell viability than 0.2 mM RA.

Yousefzadi et al. [19] demonstrated the *in vitro* cytotoxicity of the essential oil of *S. khuzistanica*, which reduced the cell viability of human colon adenocarcinoma (SW480), MCF7 and choriocarcinoma (JET3) cells. This suggests that other parts of *S. khuzistanica* plants, in this case essential oil obtained from leaves, which contain carvacrol as the main component but no RA, could have a similar anticancer activity to our biotechnological extract based on *S. khuzistanica* cell cultures, containing RA as the major component. More recently, Esmaeili-Mahani et al. [20] also reported cytotoxic activity of an *S. khuzistanica* ethanolic extract obtained from dry leaves against the MCF-7 cell line. Unfortunately, the authors did not determine the composition of the leaf extract, but they attributed the biological activity to carvacrol, being the main compound of *S. khuzistanica* aerial parts. Both RA and carvacrol have anti-proliferative activity, which may explain their similar biological effects, despite having a different phytochemical composition [4,21].

### 2.4. Induction of MCF-7 Cell Cycle Arrest by S. Khuzistanica Extracts

**Figure 5:**
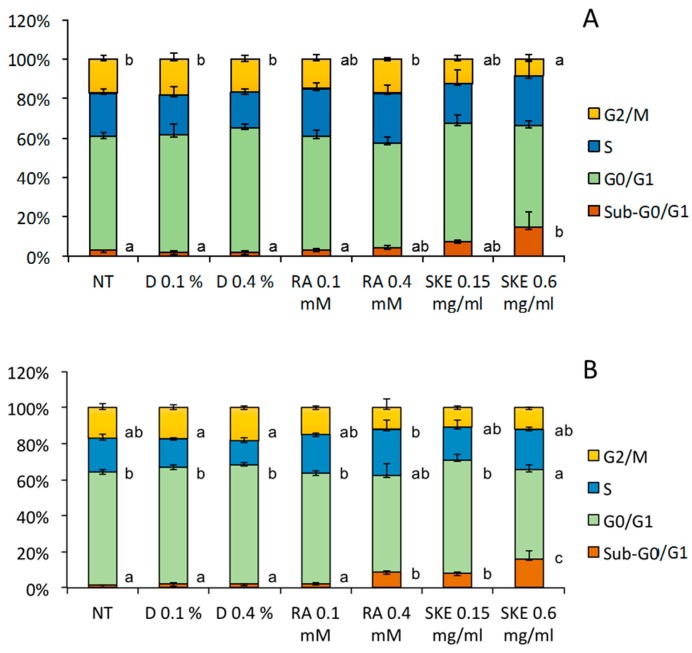
Effect of *S. khuzistanica* extracts on the percentage of MCF-7 cells in each phase of the cell cycle. Cell cycle distribution of MCF-7 cells treated for 24 h (**A**) and 48 h (**B**) with 0.15 mg·mL^−1^ and 0.6 mg·mL^−1^ *S. khuzistanica* extract (SKE), 0.1 mM and 0.4 mM rosmarinic acid (RA) or 0.1% and 0.4% DMSO (vehicle; D). Control cells were non-treated (NT). Each bar represents the mean ± SD of three replicates. For each phase of the cell cycle, different letters indicate significant differences between treatments (*p* < 0.05).

Given that SKE had a stronger impact on the viability of MCF-7 cells than HepG2 cells, and based on reports showing that RA induces cell cycle arrest in human-derived cell lines such as MCF-7 [22], the effect of SKE on the cell cycle was studied in the latter, using flow cytometry. To this end, MCF-7 cells were incubated for up to 48 h with SKE, RA or DMSO (vehicle). As previously reported, RA significantly increased the sub-G0/G1 cell population at 48 h post-treatment. Flow cytometry analysis revealed that 0.6 mg·mL^−1^ of SKE also significantly increased the percentage of sub-G0/G1 cells both at 24 h (Figure 5A) and 48 h (Figure 5B) post-treatment, and a trend to reduce the cell fractions in the G0/G1 and G2/M phases was found. Similarly as for cell viability, 0.6 mg·mL^−1^ SKE had a stronger effect on MCF-7 cell cycle than the maximum RA concentration used (0.4 mM).

### 2.5. Effect of S. Khuzistanica Extracts on Caspase Activity of MCF-7 Cells

**Figure 6:**
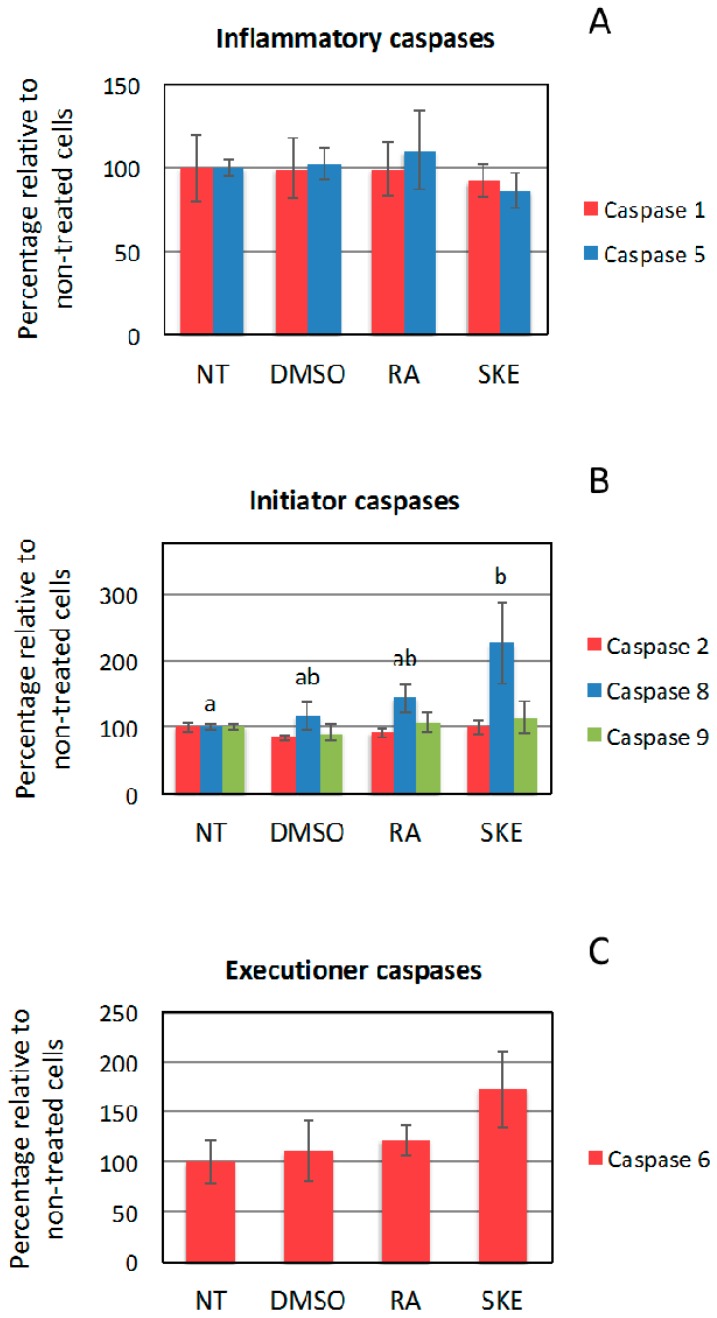
Assay of caspase activity in MCF-7 cells treated with *S. khuzistanica* extracts. The activity of inflammatory (**A**), initiator (**B**) and executioner (**C**) caspases was assayed in MCF-7 cells treated for 48 h with 0.6 mg·mL^−1^ *S. khuzistanica* extract (SKE), 0.4 mM rosmarinic acid (RA) or 0.4% DMSO (vehicle). Control cells were non-treated (NT). Each bar represents the mean ± SD of three replicates. Different letters indicate significant differences between treatments (*p* < 0.05).

The fact that induction of the sub-G0/G1 cell population is associated with cell apoptosis and that RA is known to induce apoptosis in human cancer-derived cell lines [23,24] prompted us to analyze the impact of SKE on the activity of inflammatory caspase-1 and -5, initiator caspase-2, -8 and -9, as well as executioner caspase-6 (Figure 6).

No effect on the activity of inflammatory caspases was observed at 48 h after treatment. However, compared to non-treated cells, SKE significantly enhanced caspase-8 initiator activity, which is known to mediate activation of the extrinsic apoptosis pathway [25]. Although not significant, the same trend was observed in MCF-7 cells incubated with RA. No significant effects were found on the activity of caspase-2 and -9. Regarding executioner caspases, SKE, and to a lesser extend RA, showed a tendency to increase the activity of caspase-6. Previous studies have reported that involvement of caspases in apoptosis induction by RA is cell type-specific [22]. Taken together, our findings suggest that SKE and RA may induce apoptosis of MCF-7 cells by activating the extrinsic pathway. Conceivably, ligand binding to death receptors in the cell membrane would trigger caspase-8 dimerization and activation, which in turn may lead to direct cleavage and activation of executioner caspases. However, SKE and RA failed to significantly modify the activity of caspase-9, which is the initiator caspase responsible for the intrinsic apoptosis pathway. Given that 0.6 mg·mL^−1^ SKE (containing 0.064 mM RA) had a stronger effect on MCF-7 cells than 0.4 mM RA, it may be due to the anticancer activity of other minority components of the extract, although this hypothesis would need to be confirmed by their chemical characterization.

The activation of caspase-3 by extracts of *S. khuzistanica* leaves has been previously demonstrated [20]. In the current work, this particular caspase was not studied, so we cannot affirm if our *S. khuzistanica* extract has a similar effect. As mentioned above, the plant extract is rich in carvacrol, whereas the predominant compound in our biotechnological extract was RA.

## 3. Materials and Methods

### 3.1. S. Khuzistanica Plant Cell Cultures

The experiment was performed using 125 mL flasks with a working volume (WV) of 20 mL and an inoculum PCV of 10% from a week-old *S. khuzistanica* cell suspension obtained as previously described by Khojasteh et al. [7]. As in the previous experiments, the culture conditions were as follows: T of 25 °C, 110 rpm shaking frequency and darkness. After a growth period of 14 days (near the end of the exponential phase), COR at a final concentration of 1 µM was added as an elicitor to the flasks. The culture period consisted of 21 days and samples were harvested at 0, 2, 4, 7, 11, 14, 16, 18, 21, and 24 days of growth in order to determine in-process control measurements CFW, CDW, pH, conductivity, as well as RA production. Samples were taken in triplicate and the results compared with control conditions (without elicitation).

### 3.2. Benchtop Bioreactor Scale

In order to scale up the process, a 2 L CellBag (GE Healthcare Bio-Sciences AB, Uppsala, Sweden) with a working volume of 1 L, shaken in a Khuhner orbital shaker (Birsfelden, Basel, Switzerland) in the dark at 25 °C and 35–38 rpm, with a shaking diameter of 50 mm, was used. The culture was initiated at 35 rpm and gradually the shaking was increased up to 38 rpm to obtain a good distribution of cell biomass and oxygen transfer without foaming. The sterile airflow was 0.2 L·min^−1^ and the inoculum size was 10% (*w*/*v*). In previous experiments, these conditions were determined as optimal for the growth of the cell line and to avoid out-of-phase phenomena [7]. After 11 days of culture (near the end of the exponential phase in these conditions), 1 µM COR was added. Samples were taken at days 0, 4, 7, 9, 11, 14, 16, 18 and 21. The experiment was run in duplicate.

### 3.3. In-Process Controls

PCV, CFW, CDW, pH and conductivity were determined as previously described by Khojasteh et al. [7]. In brief, for the PCV, 10 mL of cell suspension was transferred to 15 mL Falcon tubes, then centrifuged at 4000 rpm for 10 min and the PCV was read in the tube. For CFW, 10 mL of the cell suspension was centrifuged at 4000 rpm, the pellet was transferred to a tube connected to a vacuum pump for 3 min and the CFW was measured. For the CDW, frozen biomass was freeze-dried for 24 h and the CDW of each sample was registered. 

### 3.4. Rosmarinic Acid Extraction and Quantification

Extraction and quantification of RA from the samples of lyophilized cells and supernatants were performed according to Georgiev et al. [26] with some modifications as described in Khojasteh et al. [7]. In brief, 20 mg of the freeze-dried cells were suspended with 9 mL methanol. The extracts were vortexed for 2 min and incubated for 20 min in an ultrasonic bath, and then centrifuged (4000 rpm). The supernatants were evaporated under reduced pressure (vacuum evaporator, BUCHI Corporation, New Castle, Delaware, USA) at 40–45 °C, and the residue was dissolved in 1.5 mL of methanol. For cell culture extraction, 10 mL of filtered culture medium was frozen and lyophilized. The extract was dissolved in 5 mL of methanol, incubated for 24 h at 4 °C, and then filtered (0.45 µm) to remove sugars. The methanolic extracts were passed through a 0.2 μm filter. An aliquot of 20 μL of the filtrate was injected into the HPLC for RA analysis following the method previously described and validated by Sahraroo et al. [27] with some modifications [7]. The HPLC column was a Spherisorb ODS-2 (5 µm) reverse phase 4.6 mm × 250 mm connected to an HPLC-UV system ((Agilent 1100, Santa Clara, California, USA). The mobile phase A was 0.1% (*v*/*v*) formic acid solution in water and acetonitrile was the mobile phase B. A gradient system with A and B was used as follows: (0 min) 88% A and 12% B, (30 min) 80% A and 20% B, (45 min) 70% A and 30% B up to 60 min. Throughout the chromatography the flow rate was 0.1 mL·min^−1^ and the injection volume 40 µL. For calibration, an RA standard was used in various concentrations ranging from 0.5 to 200.0 μg·mL^−1^. The retention time for RA was 16 min (Supplementary Figure S1). Standard graphs were prepared by plotting concentration versus area. Quantification was carried out from integrated peak areas of the samples using the corresponding standard graph.

### 3.5. Preparation of the S. Khuzistanica Extract for Biological Assays

In order to prepare a *S. khuzistanica* methanolic extract enriched in RA (SKE), 10 g of CDW from a 24-old cell suspension treated with COR was distributed in Falcon tubes, each containing 100 mg of CDW supplemented with 40 mL of methanol. RA was extracted as described above from the tubes and the extracts were dried in a rotary evaporator. Due to the toxicity of methanol for the biological analyses, we utilized the minimum quantity of DMSO to dissolve the dry extract and obtain the *S. khuzistanica* RA-enriched extract. An aliquot of the extract was evaporated and re-dissolved in methanol for HPLC analyses. The final concentration of RA in the *S. khuzistanica* extract was 5.8 ± 0.2 mg·RA·mL^−1^.

### 3.6. Electrospray (ESI-MS) Analyses

The instrument LC/MSD-TOF (2006) (Agilent Technologies, Santa Clara, California, USA) was utilized to detect the mass of the components of the SKE. The electrospray ionization method (ESI-MS) was used in both positive and negative mode, at a fragmentation voltage of 215 V for the positive and 175 V for the negative mode, with a drying gas temperature of 300 °C, and drying gas (N_2_). Flow was 7.01 L·min^−1^, with a nebulizer pressure of 15 psi, and capillary voltages of 3.5 KV (negative) and 4 KV (positive). The mass spectra were detected in the range of m/z 100–1000. Samples were introduced into the source with an HPLC system (Agilent 1100, Santa Clara, California, USA), using the mixture of H_2_O/CH_3_CN 1:1 with a flow of 200 µL·min^−1^.

### 3.7. Human Cancer-Derived Cell Lines

Human breast adenocarcinoma-derived MCF-7 (ATCC no. HTB-22) and human hepatoma-derived HepG2 (ATCC no. HB-8065) cells were grown at 37 °C and 5% CO_2_ in Dulbecco’s Modified Eagle’s medium (DMEM) supplemented with 2 mM glutamine, 110 mg·L^−1^ sodium pyruvate, 10% fetal bovine serum, 100 IU·mL^−1^ penicillin and 100 μg·mL^−1^ streptomycin. 

### 3.8. Cell Viability Assay

MCF-7 and HepG2 cells were seeded in 24-well plates at a density of 2 × 10^4^ cells/well. Twenty-four hours later, different amounts of SKE, RA or vehicle (DMSO) were added to the cell cultures. Forty-eight hours after the addition of the compounds, cell viability was determined by means of the 3-(4,5-dimethylthiazol-2-yl)-2,5-diphenyltetrazolium bromide (MTT) assay as previously described [28]. The cells were incubated in the presence of 0.63 mM of MTT and 18.4 mM of sodium succinate for 3 h at 37 °C. Following removal of the medium, formazan was re-suspended in DMSO supplemented with 0.57% CH_3_COOH and 10% sodium dodecyl sulphate. Spectrophotometric determinations were performed at 600 nm in a Cobas Mira S analyzer (Hoffman-La Roche, Basel, Switzerland). The results are expressed as a percentage of cell survival relative to non-treated control cells.

### 3.9. Cell Cycle Analysis

Analysis of the cell cycle was performed using the PI/Cell Cycle Analysis Kit (Canvax Biotech, Córdoba, Spain). MCF-7 cells were seeded in 6-well plates at a density of 5 × 10^5^ cells/well and left to grow for 24 h. Afterwards, the cells were incubated in the presence of 0.15 mg·mL^−1^ or 0.6 mg·mL^−1^ of SKE, 0.1 mM or 0.4 mM of RA or DMSO (vehicle) for 48 h. The cells were harvested, sedimented by centrifugation (1000 rpm, 5 min), washed with ice cold PBS, centrifuged again in the same conditions and fixed in ice cold 70% ethanol for 45 min. Cells were recovered by centrifugation, washed with PBS, centrifuged again, re-suspended with 200 µL of staining solution containing propidium iodide and RNase A, and incubated at 37 °C for 30 min in the dark. The cells were analyzed by flow cytometry using a Gallios analyzer and Kaluza software (Beckman Coulter, Brea, CA, USA). The percentage of cells in each phase of the cell cycle was calculated and the apoptotic cells were considered to constitute the sub-G0/G1 cell population.

### 3.10. Caspase Activity Assays

Activity assays for caspase-1, -2, -5, -6, -8 and -9 were performed using the Caspase-Family Colorimetric Substrate Set Plus kit (BioVision, Milpitas, CA, USA). Following treatment with 0.6 mg·mL^−1^ of SKE, 0.4 mM RA or DMSO (vehicle) for 48 h, 5 × 10^6^ MCF-7 cells were harvested, sedimented by centrifugation, re-suspended in 50 µL of chilled cell lysis buffer and incubated on ice for 10 min. After centrifugation at 10,000 g for 1 min, the supernatant was recovered to assay the protein concentration and caspase activities. Total protein in cell extracts was assayed with the Bradford method [29] using BSA as a standard. For each caspase activity, the reaction mixture contained 100 µg of cell extract protein, 4.8 mM dithiothreitol and 190 µM of the corresponding caspase p-nitroaniline conjugated substrate in a total volume of 52.5 µL. The reaction proceeded at 37 °C for 2 h. Spectrophotometric determinations were performed at 30 °C in a Cobas Mira S analyzer (Hoffman-La Roche, Basel, Switzerland) at a wavelength of 405 nm (caspase activity assays) and 600 nm (total protein). The results are presented as a percentage of caspase activity relative to the control (non-treated cells).

### 3.11. Statistical Analysis

The statistical analysis was performed with SPSS version 24 (IBM, Armonk, NY, USA). Data were submitted to one-way ANOVA. Significant differences among treatments were determined with the Scheffé post hoc test.

## 4. Conclusions

Taken as a whole, our results demonstrate that plant cell cultures of *S. khuzistanica* could constitute a biosustainable source of RA and that COR is an effective elicitor for increasing its production, despite negatively affecting growth in the small-scale system. We also demonstrated that when the system was scaled up to the benchtop scale (orbitally shaken 2 L culture bag with working volume of 1 L) the negative effect of COR on biomass production was reduced and the productivity of the biotechnological system increased significantly. RA and even more so SKE reduced the viability of HepG2 and MCF-7 cancer cell lines. The fact that SKE increased the sub-G0/G1 cell population and enhanced caspase-8 initiator activity supports the notion that it activated the extrinsic apoptosis pathway in the MCF-7 cancer cell line. 

## Figures and Tables

**Table 1:** Time courses of rosmarinic acid (RA) production expressed as mg·g·CDW^−1^ and mg·L^−1^ in *S. khuzistanica* suspension cultures elicited with pre-optimized coronatine (COR) (1 µM), up to 10 days after inoculation. Each result is the average of 3 replicates ± SD.

Day	Specific Production (mg·g·CDW^−1^)		Production (mg·L^−1^)	
	Control	COR	Control	COR
**0**	67.1 ± 2.1		179.4 ± 3.7	
**2**	94.6 ± 1.9		319.3 ± 3.8	
**4**	96.5 ± 2.3		264.7 ± 4.6	
**7**	117.3 ± 2.4		312.0 ± 4.0	
**11**	128.4 ± 2.9		456.0 ± 4.9	
**14**	132.5 ± 2.7		1184.2 ± 8.6	
**16**	127.4 ± 3.1	147.3 ± 2.6	1901.8 ± 11.0	1288.5 ± 13.1
**18**	132.0 ± 2.1	195.5 ± 3.2	2121.3 ± 11.7	1873.8 ± 7.6
**21**	150.9 ± 3.2	206.2 ± 3.6	2134.0 ± 9.0	2460.8 ± 12.1
**24**	163.9 ± 3.5	221.6 ± 4.0	2221.8 ± 7.0	2665.6 ± 12.6

## Supplementary Materials

Supplementary materials can be found at https://www.mdpi.com/1422-0067/20/10/2400/s1.
