# Supplementary material for: In Vitro Study of the Anticancer Effects of Biotechnological Extracts of the Endangered Plant Species Satureja Khuzistanica
Source: Int J Mol Sci. 2019 May 15;20(10):2400. doi: 10.3390/ijms20102400 (PMC6566673; doi:10.3390/ijms20102400)
Supplement: Supplementary file 1 [file ijms-20-02400-s001.pdf]

Supplementary material:

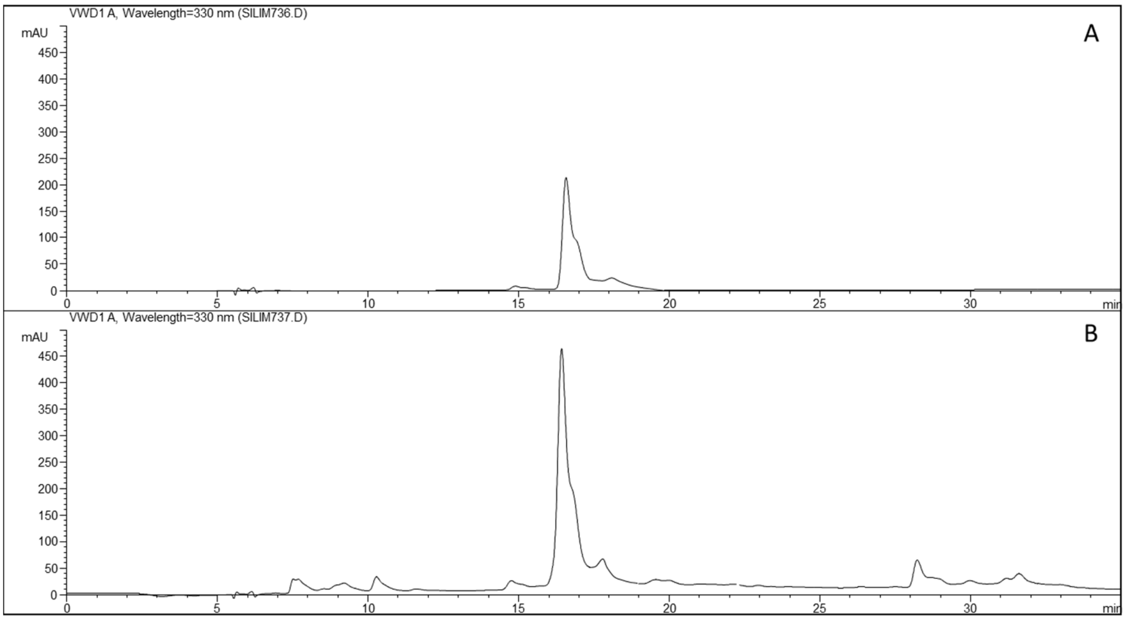

Figure 1. HPLC-UV chromatogram (330 nm) corresponding to standard rosmarinic acid (A) and a *S. khuzistanica* cell methanolic extract (B).
